# Patients´ experiences of an exercise intervention in primary care following robot-assisted radical cystectomy due to bladder cancer: a qualitative study

**DOI:** 10.1186/s12885-024-13059-y

**Published:** 2024-10-24

**Authors:** Patrik Karlsson, Malin Nygren-Bonnier, Simon Torikka, Andrea Porserud, Lars Henningsohn, Christina B. Olsson, Elisabeth Rydwik, Maria Hagströmer

**Affiliations:** 1 Division of Physiotherapy, Department of Neurobiology, Care Sciences and Society, Karolinska Institutet, Stockholm, Sweden; 2 Medical Unit Occupational Therapy and Physiotherapy, Women’s Health and Allied Health Professionals Theme, Karolinska University Hospital, Stockholm, Sweden; 3 Division of Urology, Department of Clinical Science, Intervention and Technology, Karolinska Institutet, Stockholm, Sweden; 4 Academic Primary Health Care Centre, Region Stockholm, Stockholm, Sweden; 5 Department of Health Promoting Science, Sophiahemmet University, Stockholm, Sweden

**Keywords:** Abdominal surgery, Cancer survivorship, Oncology, Physical activity, Physiotherapy, Postoperative complications, Rehabilitation, Thematic analysis, Surgery

## Abstract

**Background:**

Physical activity is thought to be a key component in reducing postoperative complications following major abdominal surgery. The available literature on exercise interventions following radical cystectomy in patients with bladder cancer is scarce but suggests that physical activity and exercise might improve physical function and health-related quality of life, thus calling for further investigation. The CanMoRe-trial is a single-blinded randomised controlled trial (Clinicals Trials NCT03998579 25/06/2019), aimed at evaluating the impact of an exercise intervention in primary care following robot-assisted radical cystectomy. This study seeks to explore patients’ experiences of the exercise intervention in the CanMoRe-trial to gain a better understanding of facilitating aspects and potential barriers.

**Methods:**

A qualitative study was conducted involving 20 patients from the intervention group of the CanMoRe-trial who were interviewed individually between October 2020 and March 2023 using a semi-structured interview guide. The interviews were recorded and transcribed verbatim and reflexive thematic analysis was used to analyse the data.

**Results:**

Four main themes were identified: *Having to adapt to new circumstances*, describing the challenges regarding physical activity patients face after discharge. *Optimising conditions for rehabilitation*, describing how practical conditions affect patients’ ability to exercise. *Motivated to get back to normal*, describing patients´ desire to get back to normal life and factors influencing motivation. *Importance of a supportive environment*, describing the impact of social support, support from physiotherapists, and how the environment where exercise takes place impacts patients’ ability to exercise.

**Conclusion:**

This study found that patients participating in the CanMoRe-trial are positive towards physical exercise in PC following radical RARC. They are motivated to get back to normal life but face major challenges when arriving home following surgery, which affect their ability to perform physical activity and engage in exercise. Conditions need to be optimised to support patients’ ability to engage in exercise by providing an accessible PC location to perform exercise in. A supportive environment is also needed, including guidance from healthcare professionals regarding which type of exercise, intensity and amount of exercise that should be performed, enabling patients gradually to develop self-efficacy regarding exercise and focusing on goals related to patients’ normal lives before surgery.

**Supplementary Information:**

The online version contains supplementary material available at 10.1186/s12885-024-13059-y.

## Introduction

Radical cystectomy is considered the golden standard for treatment of muscle-invasive bladder cancer, often combined with neoadjuvant chemotherapy [1]. Such surgery is increasingly performed via Robot-Assisted Radical Cystectomy (RARC) [2, 3]. The surgery includes urinary diversion by either an orthotopic neobladder or an ileal conduit with a urinary stoma [1]. Complications following RARC are common and 19-75% of patients must be readmitted to the hospital following surgery due to complications [4]. Common complications include infection and thromboembolic or cardiac complications [5]. The risk of complications and premature mortality seems to be associated with the fact that patients undergoing RARC are often older and have comorbidities [1]. Physical activity is thought to be a key component in reducing postoperative complications following major abdominal surgery and is often a highly recommended intervention in perioperative care protocols [6, 7].

There is strong evidence to support that physical exercise among cancer survivors mitigates symptoms and treatment-related side effects, such as anxiety, depressive symptoms and fatigue, and also improves physical function and health-related quality of life [8]. Patients diagnosed with cancer are recommended to follow the general guidelines of weekly physical activity for at least 150 min of moderate intensity, or 75 min of vigorous intensity, in combination with at least two weekly sessions of strength training consisting of at least two sets of 8–15 repetitions of an intensity of at least 60% of one repetition maximum [8–10]. Higher levels of physical activity are also shown to decrease both all-cause mortality and cancer-related mortality in cancer survivors [9]. However, there is evidence suggesting that patients with cancer are less likely to be physically active, or change their physical activity behaviour following diagnosis, calling for strategies to support physical activity in cancer survivors [11]. Patients with bladder cancer are no exception and a recent study of 936 patients shows that one third self-reported a sedentary lifestyle and poor physical health [12]. The study also concludes that implementation of exercise interventions could improve the health status of patients with bladder cancer and that clinicians should try to get patients engaged [12].

The CanMoRe-trial is a single-blinded randomised controlled trial aimed at evaluating the impact of an exercise intervention in primary care (PC) following RARC [13]. One aspect of evaluating an intervention is trying to understand how the intervention interacts with the context in which it is implemented and with the individuals involved [14]. Further, it is important to investigate user experiences and facilitating aspects and barriers that influence implementation as a guide for potential further implementation of an intervention [15]. This study seeks to explore patients’ experiences of an exercise intervention in PC following RARC to gain a better understanding of facilitating aspects and potential barriers related to the CanMoRe-trial intervention.

## Methods

### Design

A qualitative study using individual semi-structured interviews was carried out, allowing for probing questions and exploration of patients’ narratives during the interviews. Thematic analysis was used to analyse the data, with application of an inductive approach. The COREQ 32 item checklist was used as a guide when producing this report [16].

### Context

Recruitment to the CanMoRe-trial was carried out at the Karolinska University Hospital in Solna, Sweden from August 2019 to October 2022 [13]. Patients scheduled for curative RARC due to bladder cancer were eligible for inclusion if they could walk with or without a walking aid, understood Swedish in speech and writing, had no documented cognitive impairment, and lived in Stockholm County. Included patients were admitted to the urology ward the day before surgery. After surgery they spent one night in a post-operative recovery ward before returning to the urology ward. At the end of their hospital stay patients were randomised to either a control group or the intervention group in the CanMoRe-trial [13]. Patients in the intervention group received a referral to a PC clinic near their home, with appointments with a physiotherapist scheduled for twice a week for 12 weeks. At the PC clinic, patients followed a physical exercise programme consisting of general resistance training and aerobic exercises (Supplementary Material 1) Patients received information that the program had been approved by the surgeons and that individual adaptation was allowed and guided by a physiotherapist. and that the programme was also progressive in relation to patients’ postoperative restrictions. Patients in the intervention group were also encouraged to perform daily walks and a home-based programme of pelvic floor and abdominal exercises to lower the risk of stoma hernia [13]. Patients from the entire Stockholm County in need of RARC are operated on at the Karolinska University Hospital in Solna, making the potential geographical spread of patients’ homes great. Therefore, 18 PC clinics were recruited to participate, scattered across Stockholm County to be able to offer all patients a PC clinic within reasonable distance from their home.

### Sampling strategy

A purposive sampling strategy was used. Patients allocated to the intervention group in the CanMoRe-trial, i.e., exercise in PC, were asked to participate in this interview study. When signing the informed consent form for the CanMoRe-trial, patients were also asked if they could be contacted for a potential interview following the exercise intervention; no patient declined to be contacted. Upon completion of the exercise intervention, patients were contacted by either author PK or author ST by telephone and invited to participate in this interview study. Patients who agreed to participate in the interview study were then scheduled for an individual interview; 23 patients were asked to participate and 20 accepted.

### Data collection

The interviews were conducted individually by ST (Male, RPT, MSc) between October 2020 and March 2023 using a semi-structured interview guide developed by the authors for this study (Supplementary Material 2), allowing for probing questions. Each participant was interviewed once. Due to the Covid-19 pandemic and logistical reasons, interviews took place a mean of 199 days (min-max 0-305 days) following completion of the PC intervention and were mainly conducted at a distance: by telephone (*n* = 2) and digital meeting platform (*n* = 12); some interviews were conducted face to face at a place of the patient’s choosing (*n* = 6). Interviews lasted between 34 and 82 min (mean 46) and were recorded using digital software when conducted via digital meeting platforms, and by a recording device in the rest of the interviews. The interviews were then transcribed verbatim: ST transcribed nine interviews, PK (Male, RPT, MSc) transcribed seven interviews, and four interviews were transcribed by a professional agency. A pilot interview was conducted and included in the study as no major changes were made.

### Data analysis

Reflexive thematic analysis, as described by Braun and Clarke, 2006, was used to analyse the interviews [17]. Author PK followed the six-step process of: Familiarisation with the data: reading, and re-reading the transcribed interviews; Generating initial codes: marking interesting sections of the data and making initial notes on sections that connect to the aim of the study throughout the data set; Searching for themes: organising codes into preliminary themes and gathering all data relevant to the themes; Reviewing themes: checking if the themes work in relation to the initial codes and notes throughout the data set and generating a schematic overview of the analysis and themes; Defining and naming themes: refining the themes and generating a clear definition and name for each theme; Producing the report: extracting examples and quotations for the report and reviewing their stringency throughout the data set and in relation to the aim of the study [17]. Using research triangulation to enhance trustworthiness, author ST also analysed several interviews, after which PK and ST held a comparative discussion before agreeing on the themes. Authors MNB (Female, RPT, PhD) and MH (Female, RPT, PhD) also took part in the process by reviewing the work of PK and ST throughout the analysis. The definition of themes and names was carried out jointly by authors PK, ST, MH, and MNB to ensure that there was stringency in the connection between results and data.

## Results

**Fig. 1:**
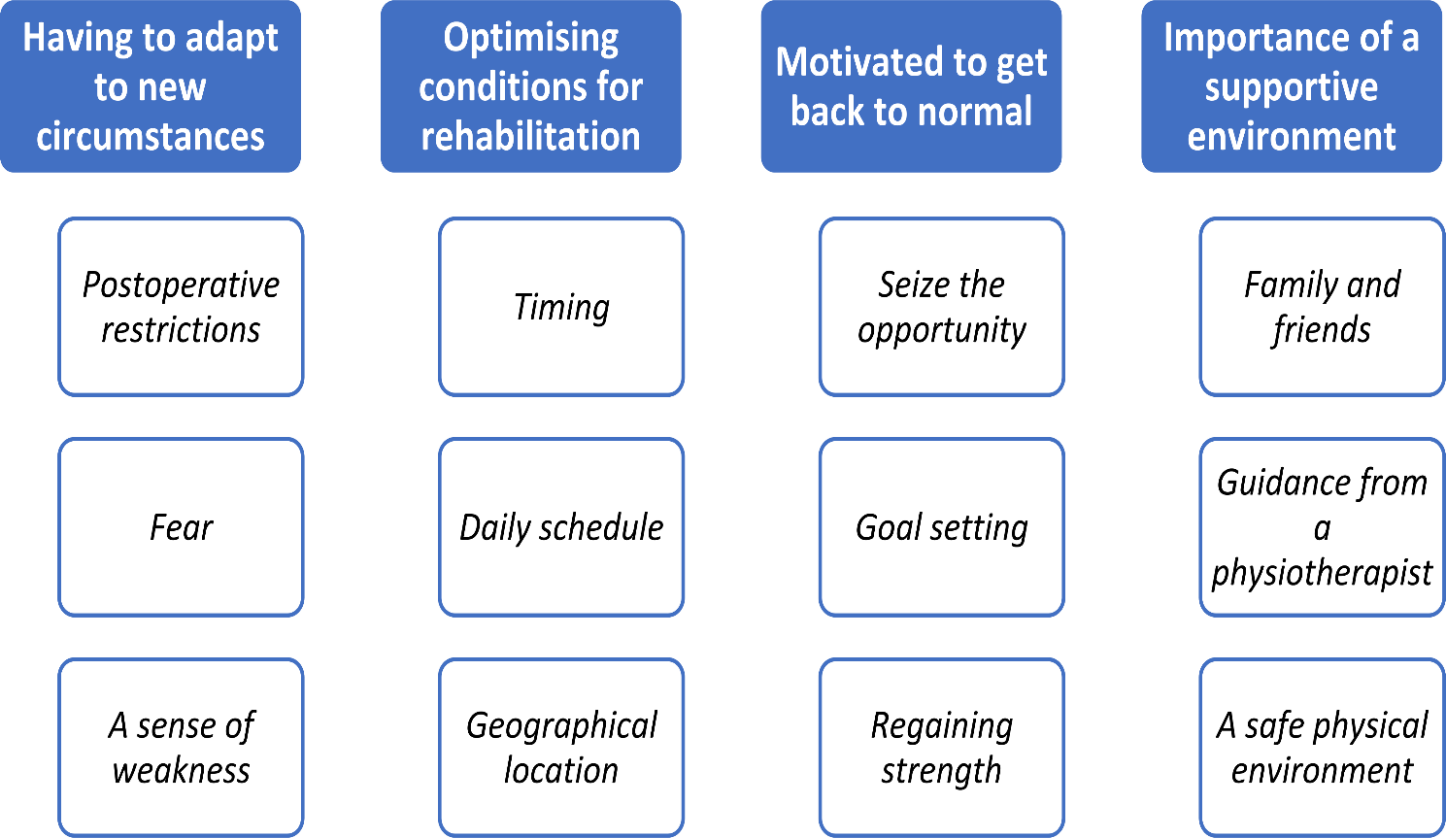
A schematic overview of the main themes and their related sub-themes

A short description of the basic characteristics of the interviewed patients can be found in Table 1. Four main themes were identified, and an overview of the main themes and related sub-themes can be seen in Fig. 1.

**Table 1:** A description of demographic and clinical characteristics of participants, *n* = 20

Characteristics	
Sex, n (%)	
Male	14 (70)
Female	6 (30)
Age, years	
Mean (range)	70 (56–82)
Family status, n (%)	
Married/living together	12 (60)
Living alone	8 (40)
Working status, n (%)	
Working	6 (30)
Sick leave	2 (10)
Retired	12 (60)
Neoadjuvant therapy, n (%)	
Yes	5 (25)
Urinary diversion, n (%)	
Orthotopic bladder	1 (5)
Ileal conduit	19 (95)
Length of stay, days	
Mean (sd)	8 (2.9)
Start PC after discharge, days	
Mean (range)	38 (10–93)
Number of PC sessions*	
Mean (range)	18 (8–24)

*Number of sessions in PC out of 24 possible

### Having to adapt to new circumstances

On returning home from the hospital, patients describe a feeling of having to adapt to new circumstances following surgery and treatment. They describe how their body is not what it used to be; there is a discrepancy between previous physical ability and function and the current situation, and initially this is almost a shock or a crisis. Patients feel uncertain about whether they will ever get better and whether rehabilitation will really be possible.

#### Postoperative restrictions

Patients describe having to make adjustments and adaptations to their daily life and routines due to restrictions following surgery. Having to refrain from strenuous activities such as carrying heavy loads prevents patients from doing daily chores like grocery shopping and gardening. Patients report a new level of need for help from other people and a lowered sense of independence as they cannot do what they used to. Not being able to work or participate in leisure sports due to the new circumstances and restrictions is also felt as a hard transition. Patients must also adapt to having a urostomy and to the shots of medication that are initially necessary after surgery to lower the risk of complications; these are experienced as worrisome in relation to physical activity and exercise.

*“Yes*,* well of course that’s obvious*,* you have had an operation*,* let me think*,* some difficulties. Well*,* you can’t push yourself*,* there are of course many things you can’t do*,* you also become very restricted really when you have had a stoma operation” – Interview 6 (Male*,* age 63)*.

*“I suppose I pushed myself a bit too much and I am paying for that today*,* I suppose I got a bit*,* you sort of didn’t understand how fragile you are really when they have cut your belly” – Interview 5 (Female*,* age 80)*.

#### Fear

Patients express an initial feeling of fear due to their new circumstances. They fear complications if they do not adhere to the rehabilitation regime and comply with the general recommendations regarding physical activity following surgery. However, they also fear complications if they exert themselves, lift things that are too heavy or push their physical ability too far. Patients express a fear that if they do too much, their abdomen will not hold up and their surgical wounds might burst. They also express a fear of hernias and that the urostomy bag might burst or leak during exercise. Patients describe a feeling of not knowing how hard they can push themselves and that this results in a fear of movement, hindering both their daily physical activity and their exercise. Patients also say that fear of pain and discomfort make them adapt daily life and physical activity. Patients without a urostomy express a fear of urine leakage in public, constantly feeling the need to know where the nearest bathroom is, not knowing when they might need to go. Some also express a fear of public places and avoid them altogether initially due to shame of their urostomy or inability to control their bladder. Patients also describe a constant underlying fear of recurrence of cancer, sometimes influencing their daily lives and ability to focus on other things.

*“But I suppose I was a bit scared that*,* well… So*,* then I took it a bit easier than what I did before the operation. Without having had any experience of how much I could stress this bag (the ostomy)” – Interview 1 (Male*,* age 77)*.

*“Of course*,* you were very scared that the operation would so to speak fall apart. That those ends that they cut together and made a new connection with the kidneys*,* of course you were scared that all that would break and create further problems. So*,* you had to be very careful with everything. I couldn’t lift things or work in the garden that I normally do. And all the time you were scared that something would break somewhere in*,* in the belly*,* seeing that it had been stitched together” – Interview 2 (Male*,* age 78)*.

#### A sense of weakness

On arriving home following the surgery patients express a sense of weakness, fatigue, and listlessness. They describe having lost a lot of their previous strength and vigour, barely managing daily life activities, and feeling exhausted. Some patients say that they do not recognise themselves, not being able to do or enjoy things as they used to. Some patients state that they could not have imagined the state they would be in now following surgery despite having had good physical fitness prior to surgery. That they could be so badly affected after surgery comes as a shock: losing both strength and cardiovascular ability, feeling out of breath after short walks, not being able to manage to work or to play with grandchildren. Some patients who have had multiple treatments, such as chemotherapy, prior to surgery express despair about their loss of strength and body weight, as well as their desperate need to regain physical function and strength.

*“Of course*,* it was quite bad*,* that’s how it felt. And naturally it felt very far away*,* to get back in shape so to speak.”- Interview 16 (Male*,* age 57)*.

*“I was really so exhausted when I got back home that I took midday naps*,* I sort of never do that normally*,* but I took midday naps and so on. I felt fairly weak when I got back home” – Interview 4 (Female*,* age 64)*.

### Optimising conditions for rehabilitation

Patients describe the importance of tailored and optimised practical conditions for providing the best possible chance of getting started and following through with their rehabilitation regime. Getting a referral to a PC clinic makes it easier to get started as they might otherwise postpone rehabilitation or exercise due to their new circumstances and not feeling well enough.

#### Timing

The timing of the start of rehabilitation following surgery is described as sensitive, with a delicate balance between getting started early and getting started too early, as some recovery time is needed. Patients want to start the rehabilitation process early following surgery as they are eager to regain strength and function, but to do this they need support and want contact with PC shortly after getting home from the hospital. The idea, according to the patients, is that the earlier you start, the faster you will get back on track and back to normal. However, they are also aware of needing a short time between arriving home from the hospital and the actual start of rehabilitation within PC. Immediately after surgery and arrival home, patients experience an overwhelming feeling of fear and a sense of weakness, as described earlier. The idea of starting physical rehabilitation immediately is described by the patients as daunting and almost impossible. Letting a week or two pass between arriving home and the start of rehabilitation within PC, allows patients to adjust to the situation as well as to observe specific aspects or weaknesses which might need special focus during the rehabilitation.

*“Well*,* it was*,* it was that I felt so unwell early on that I don’t think I could have started any earlier*,* to be honest*,* so it was more or less right.” – Interview 9 (Male*,* age 57)*.

*“It was definitely not too early*,* not for me at any rate*,* I might perhaps have started even earlier.” -Interview 8 (Male*,* age 80)*.

#### Daily schedule

Having two rehabilitation sessions booked per week for 12 weeks in a PC clinic was described as something that needed to be fitted into the patients’ daily schedule. For most patients the notion of having a booked appointment at the PC clinic was seen as positive and important – it provides a structure and makes it harder to postpone or skip the rehabilitation session. But fitting it into the daily schedule is not always easy. In addition to work and daily life, patients also have several follow-up appointments at the hospital and other healthcare institutions after surgery. This makes it hard to fit in two rehabilitation sessions per week as well, especially if the timing of these sessions is not somewhat flexible. Patients who are not working but are on sick leave or retired describe having no problems fitting the rehabilitation sessions into their schedule. The patients’ descriptions also showed a difference in how the different PC clinics managed the physiotherapist sessions. Some were more flexible depending on their own needs and planning. Some PC clinics, on the other hand, seemed to have set times during each week when a patient was welcome for supervised exercise, forcing the patient to adjust their schedule to that of the clinic.

*“Of course*,* I was off sick as well*,* so I didn’t work then so to speak. That made it quite easy for me and you are sort of free*,* you can just go there.” – Interview 10 (Male*,* age 76)*.

*“About this training*,* it was probably more about fitting it in with work*,* you don’t get time off to do this because it wasn’t on a doctor’s prescription but a project.” – Interview 12 (Female*,* age 56)*.

#### Geographical location

Patients state that the location of the PC clinic where the physical rehabilitation takes place is an important aspect of making it easier to start and follow through with the rehabilitation period. Feeling weak and unwell, and having a lower level of physical ability than previously, makes it hard to travel often and over long distances. Long journeys are described as being both physically and mentally challenging and this makes it easier to find excuses not to go. Having a short distance to travel saves energy which can then be spent on exercise instead of travel, and it also makes it easier to attend even if one is feeling a bit unwell that day. Patients also draw attention to other specific practical aspects of having the PC clinic nearby, such as that this makes it easier to plan toilet visits and emptying or replacing the urostomy.

*“But then of course you arranged it so that I had one right here in Solna*,* right across from where I live? But if I had had to travel and – council transport or something like that? Then I think would have hesitated.” – Interview 15 (Male*,* age 74)*.

### Motivated to get back to normal

Patients report that they are highly motivated to start rehabilitation to regain their strength, endurance and energy, and to get back to normal life and be able once again to perform activities in daily life independently and to get back to work and leisure activities. But patients emphasise the importance of getting support in this process, since they not only have a weakened starting point but also feel insecure regarding which level of activity and exercise is correct and safe.

#### Seize the opportunity

Patients interviewed in this study express gratitude for the opportunity to exercise and rehabilitate with the support of a physiotherapist in PC. They describe feeling motivated to seize this opportunity to get professional help and support with their rehabilitation. If it had not been for this possibility, they believe that it might have been a lot harder to self-motivate and start physical rehabilitation. Patients also observe that the awareness that professional help and support is available for a limited period of 12 weeks helps them to prepare mentally and makes them motivated to put in the effort to prioritise rehabilitation during this period to get the most out of it.

*“It was great to be given this chance and I felt that the rehabilitation would be much better if I sort of got a bit of help and a bit of a push*,* eh*,* I think it was afterwards*,* when I felt so awfully bad after the operation*,* those first weeks*,* then it felt really good to be given this support that I was to get.” – Interview 9 (Male*,* age 57)*.

#### Goal setting

Patients describe that their ultimate goal for the rehabilitation is to recover their previous level of physical function and wellbeing. They consider goals linked to the exercise itself, such as increasing the number of daily steps or increasing the resistance set for a specific exercise, feel less relevant than goals linked to everyday life. While goals increase motivation, it is hard to focus on any other goal then the overarching one of getting back to normal. The goals that provide the strongest motivation are things like getting back to work, or engaging in hobbies or sport activities, playing with grandchildren, or looking after the garden. With goals like these in mind, patients feel a strong motivation to follow through with their rehabilitation.

*“Of course*,* I was so super keen because I wanted to get back to a normal life. We are fairly active*,* we go cycling and we*,* well if it is in winter then we go skiing*,* I have done a lot of skiing and been in the mountains a lot and things like that*,* go for walks a lot and walk in the woods and of course I wanted to get back to that.” – Interview 3 (Male*,* age 71)*.

#### Regaining strength

Rehabilitation is described as a tool for regaining strength, endurance and energy. Patients feel motivated since they know that rehabilitation will help them improve their physical abilities and make them feel well again. They describe how this feeling increases during the rehabilitation period as the results of their efforts gradually make themselves felt. Seeing and feeling progress during the rehabilitation period adds to the motivation; being able to increase the intensity and resistance of the exercise gives a sense of feeling strong again and able to manage and accomplish something. Gradually regaining strength and endurance does not only lead to progress in the gym but also in daily life and patients describe how rehabilitation helps them manage and overcome difficulties that they initially experienced following surgery due to reduced physical ability. Patients regard exercising the entire body through resistance training in combination with endurance training as something positive, since it helps the recovery of general physical function and not just aspects specific to the post-surgical abdomen. The feeling of gradually regaining strength during the rehabilitation period also encourages patients to be more active in their daily life and to increase their physical activity and it even motivates them to continue exercising on their own after the rehabilitation period has ended.

*“The training programme has been a great help for me so to speak. It meant that directly after*,* directly after midsummer*,* just coming*,* just coming out of hospital and getting going*,* then it makes it easier to walk and easier*,* easier to exert oneself*,* and easier to do all the things that you normally do*,* in the garden and look after it and so on.” – Interview 8 (Male*,* age 80)*.

*“And I will say there is a colossal difference if I do the same exercises now compared to what it was like at the beginning*,* that I will say*,* hah*,* back then I could hardly manage anything. Now it is dead easy to do the same thing.” – Interview 9 (Male*,* age 57)*.

### Importance of a supportive environment

Based on their experience during the rehabilitation period, and considering their circumstances, patients describe the importance of having adequate support if they are to be able to exercise and follow through with their rehabilitation. Getting started with exercise and rehabilitation following surgery is a challenge as previously described, and the patients are clear that a supportive environment helps them overcome this.

#### Family and friends

Social support from family, friends and coworkers is described as a major factor in overcoming the challenges of getting started and following through with rehabilitation, as well as the challenges of daily life following surgery. Patients describe how such support makes it easier to get started and making the effort to go to the PC clinic, even though it might feel hard some days. Patients also describe how encouragement from family and friends helps keep the motivation up, partly because they want to be strong for their loved ones, but also not wanting to disappoint them. Being open to family and friends about the situation, such as having feelings of weakness or issues with having a urostomy, is also described as an important route towards better understanding and further support from one’s social environment.

*“But now I have the good luck*,* I have of course*,* my wife is of course*,* so of course she has helped me masses of course*,* you can’t deny that*,* and I am bloody grateful for that*,* so that*,* yes.” – Interview 6 (Male*,* age 63)*.

#### Guidance by a physiotherapist

Support from a physiotherapist when getting started with exercise and rehabilitation following surgery is of major importance according to the interviewed patients. Some patients describe themselves as having limited or no experience of either exercise or rehabilitation, which further emphasises the need for support by a professional to get started. But even patients with extensive experience of exercise, fitness and participation in sports express the need for support by a professional when starting rehabilitation following surgery. Patients state that they need guidance regarding relevant exercises, their intensity, and progression. Because of their situation, patients fear straining themselves or exerting themselves too much, possibly resulting in complications as previously described. With support they feel safe and gradually build trust in the physiotherapist, which then enables the patients to push beyond their fear and exert themselves at an adequate level and thus gradually improve their physical abilities. Guidance from a physiotherapist was also seen as helpful for progressing in a safe manner, as patients sometimes need a push, and sometimes need to be held back. It was important to the patients that the physiotherapist understood their situation; this would strengthen the trust felt and enable patients to gradually challenge themselves more. The physiotherapy guidance was described as especially important when dealing with complications or other ailments, and for receiving help with individual adaptation of the rehabilitation programme when issues such as pain, discomfort or urostomy problems emerged. Having continuous contact and support from a physiotherapist was also described as a factor supporting adherence with the rehabilitation regime.

*“I developed trust in him. Because what happened was that a machine right at the beginning*,* I felt it was too heavy for my legs there. It felt like*,* eh… it wouldn’t… the bag wouldn’t stay put and so on. So*,* then he said*,* then you take it a bit easier. So*,* I felt complete trust in him” – Interview 1 (Male*,* age 77)*.

*“But I definitely think that this rehab played a big part. That you got going and moving. Otherwise*,* it would no doubt be easy just to do nothing. Sit there and go oh dear*,* oh dear. Do I have the strength for this? But because I was doing things down there at the rehab*,* under supervision*,* then of course I noticed that I am not as locked down as all that. I can – there is quite a lot that I can do.” – Interview 11 (Male*,* age 63)*.

#### Physical environment

Given the patients’ circumstances and often limited experience of exercise and rehabilitation, fitness centres seemed uninviting and unsuitable. Instead, the interviewed patients expressed a need for a more supportive environment adapted to their needs. Being able to perform their rehabilitation in the safe environment of a PC clinic with healthcare professionals and other patients present, makes them feel comfortable and encouraged. When you are frail or feel weak, exercising among other individuals in similar situations is described as making the experience less dramatic and the patient more confident regarding exercise. Some patients observe that because of their urostomy they would feel uncomfortable or ashamed in a fitness centre while a PC clinic feels more suited to patient rehabilitation. Patients also state that exercising together with other patients is stimulating as well as enabling them to support each other, even if their rehabilitation need is due to different ailments.

*“I have had a bit of a prejudice seeing these big gyms with mirrors and windows*,* fitness freaks*,* but here I was sitting with the lame and the crippled and old women*,* didn’t I*,* I am trying to carry on just sitting there cycling*,* and then there is an old biddy beside me*,* 80 plus*,* cycling*,* so when I sort of look at people like this*,* well but*,* all of us had had operations or had some problem*,* it is just human*,* it wasn’t a competition or smooth and shiny naked chests.” – Interview 12 (Female*,* age 56)*.

## Discussion

This study explores patients’ experiences of an exercise intervention in PC following RARC due to bladder cancer. It aims to reach a better understanding of facilitating factors and potential barriers to exercise following RARC. The results yielded four themes illustrating what may affect patients’ ability to engage in exercise in PC following RARC. There are only limited studies exploring experiences, attitudes and preferences regarding physical activity and exercise in patients with bladder cancer and following radical cystectomy, but these largely agree with the results of this study [18].

In the present study, patients describe how they must adapt to their new circumstances and the challenges they face in their daily life after arriving home. Internal factors such as these that affect patients’ ability to engage in exercise have also been noted in a study exploring physical activity behaviour before and after radical cystectomy, which also found that patients need time to adjust after arriving home following surgery, that they struggle with the new circumstances, and that this affects their ability to perform physical activity. The same study also found that fear of leakages, and of putting too much pressure on the urostomy, also affects patients’ willingness to perform physical activity [18], thus confirming the findings of our study. This indicates that patients might benefit from help by healthcare professionals to get started with physical activity and exercise following RARC. The challenges patients face should be taken into account when planning and implementing rehabilitation or exercise interventions following RARC. The results also support the notion that patients should engage in physical activity and exercise following cancer treatment as this has been proven to mitigate some of the negative aspects of such treatment experienced by the patients, such as fatigue and weakness [8, 10].

Given the situation of the patients, providing the optimum practical conditions is important if rehabilitation is to be possible. This represents an external factor that may affect patients’ ability to engage in exercise following RARC. The timing of the start of rehabilitation appears to be important, with patients eager to start as soon as possible to proceed in their rehabilitation process. But they also express that starting to early can be both discouraging and hard. Even though the ambition was for patients to start exercise as early as two weeks after discharge, there were a number of factors affecting patients’ ability to start such as complications, not feeling well enough, in-patient rehabilitation visits and issues with the urostomy. Thus, to address patients desire to start exercise early but still give time before start in PC, an exercise program could include prescribed walks initially to bridge the gap. This is confirmed by the results of a questionnaire study of 397 bladder cancer survivors that explored exercise programming and counselling preferences. This study found, for example, that 39% of patients would prefer to start rehabilitation immediately after treatment [19]. Having access to a PC clinic within a short distance seems to be a facilitating aspect, both increasing the patients’ motivation to attend and reducing the travel difficulties linked to the patients’ circumstances. In a previous pilot study, we explored the impact of a physical exercise intervention following RARC that was carried out at the hospital. This intervention improved patients’ functional ability and aspects of their health-related quality of life, but the high drop-out rate showed that it was not practicable to perform the intervention at the hospital as travel distances were too great [20].

Patients in our study describe their strong motivation to get back to normal and regard rehabilitation as a way of achieving this – an internal factor influencing patients’ attitude to exercise. Another study also describes the desire to get back to normal life, to return to participation in sport or other activities, and not to be perceived as sick, as motivation for physical activity [18]. In that study as well as in ours, patients describe their gradually increasing self-efficacy resulting from the positive effects of physical activity and exercise, which further encourages such behaviour. Self-efficacy has also been shown to be one of the main predictors for positive physical activity in cancer survivors [21]. This suggests that gradually building patients’ self-efficacy regarding physical activity and exercise and focusing on goals related to their normal life before surgery could be key elements in facilitating exercise following surgery.

A supportive environment is highlighted as an important external factor influencing patients’ ability to perform exercise in PC following surgery. In this study, patients describe the importance of guidance from a physiotherapist both to get started with the exercise and to exercise at an appropriate level –sufficient to get al.l the benefits of exercise, but not so much as to suffer complications. Patients in our study also state that it is beneficial to be given an appointment and visit a PC clinic, provided it is reasonably close to their home. On the other hand, Karvinen et al. found that a majority of patients (53.7%) would prefer to do their exercise at home [19], which runs counter to the results in our study. However, they also found that patients prefer to receive face-to-face exercise counselling from exercise specialists and preferably at a cancer centre, which to some extent confirms our findings [19]. Rammant et al. also found patients to be less afraid of resuming their daily life activities and more confident about being active if they receive support from healthcare providers regarding suitable activities following surgery [18]. This further suggests that guidance by healthcare professionals is needed to facilitate exercise following surgery. Social support from friends and family also seems to play a part in supporting patients through their rehabilitation process. Family caregivers often play a major part in patients’ recovery, but they need to receive targeted information to be able to support the patient more effectively [22]. The need of support from health professionals regarding exercise found in our study has also shown in studies including patients with other types of cancer. When exploring the rehabilitation needs of colorectal cancer survivors, Yi et al. found that the patients also require support and information from healthcare professionals in order to both understand the importance of exercise, reduce the fear of complications due to exercise, and gain motivation to exercise [23]. Patients also require specific instructions on how to exercise adequately in regard to type of exercise, intensity and amount of exercise [23]. Furthermore, they also found that gaining support from family and fellow survivors can help increase motivation and ability to exercise as part of the rehabilitation [23]. This also aligns with our results and indicates that family caregivers should be included when information regarding exercise is given to the patents.

### Methodological considerations

Individual interviews, guided by a semi-structured interview guide, were used to make it possible to dive deeper into each patient´s individual experience. Previous research has indicated that individual interviews might generate a broader list of topics within a domain compared to focus groups [24]. Of the 23 patients who participated in the intervention, 20 were interviewed, leaving a total of three patients’ experiences unexplored. The sample included in this study is assumed to have high information power due to the patients’ personal experiences and the quality of the dialogue. This supports the assumption that the interviewed sample of 20 patients should be sufficient for the aims of the study [25]. However, a major limitation is the fact that only patients who completed the PC intervention and seem to have a positive attitude towards it, chose to participate in this interview study, thus introducing selection bias and leading to missing perspectives. This is unfortunate as gaining the perspectives of patients who dropped out of the PC intervention or chose not complete would be highly valuable as it could shed light on aspects that might need to be improved in the intervention. Furthermore, the interviews took place long after the PC intervention, which potentially limited the accuracy of patients’ recollections and introduced recall bias. Another limitation is that no information regarding patients’ previous exercise experience was collected, such as type of exercise, settings or amount of exercise that patients had performed as this could impact patients’ attitudes towards exercise.

The design of this study included measures to enhance its trustworthiness, as described by Shenton, 2004 [26] and summarised in relation to thematic analysis by Nowell et al., 2017 [27]. To avoid bias during translation of the quotes supporting the results, a professional agency was used for translation. The study was planned and designed by members of the research group who had experience of qualitative methodology (PK, MNB). An initial pilot interview was also conducted to confirm the validity of the interview guide. The interviewer (ST) had no previous engagement with the CanMoRe-trial or with the patients involved, but had clinical experience of patients following abdominal surgery, which limited bias while ensuring contextual knowledge [26]. Researcher triangulation was also used during the analysis process: separate analyses of various interviews were carried out and the results compared, with a high level of confirmability [27]. As the study was conducted over a long period of time, the interviews were transcribed by two authors, possibly introducing bias, but also strengthening the alignment when discussing the analysis in the research group.

## Conclusion

This study found that patients participating in the CanMoRe-trial are positive towards physical exercise in PC following radical RARC. They are motivated to get back to normal life but face major challenges when arriving home following surgery, which affect their ability to perform physical activity and engage in exercise. Conditions need to be optimised to support patients’ ability to engage in exercise by providing an accessible PC location to perform exercise in. A supportive environment is also needed, including guidance from healthcare professionals regarding which type of exercise, intensity and amount of exercise that should be performed, enabling patients gradually to develop self-efficacy regarding exercise and focusing on goals related to patients’ normal lives before surgery.

## Electronic supplementary material

Below is the link to the electronic supplementary material.

Supplementary Material 1

Supplementary Material 2

## Data Availability

The datasets generated and/or analysed during the current study are not publicly available due to it being qualitative data in Swedish, but are available from the corresponding author on reasonable request.

## Abbreviations

RARC: Robot-assisted Radical Cystectomy
PC: Primary Care

## References

[1] Witjes JA, Bruins HM, Cathomas R, Compérat EM, Cowan NC, Gakis G, et al. European Association of Urology Guidelines on muscle-invasive and metastatic bladder Cancer: Summary of the 2020 guidelines. Eur Urol. 2021;79(1):82–104. 32360052 10.1016/j.eururo.2020.03.055

[2] Gul ZG, Katims AB, Winoker JS, Wiklund P, Waingankar N, Mehrazin R. Robotic assisted radical cystectomy versus open radical cystectomy: a review of what we do and don’t know. Translational Androl Urol. 2019;10(5):2209–15. 10.21037/tau.2019.11.32 PMC8185680 34159104

[3] Lobo N, Thurairaja R, Nair R, Dasgupta P, Khan MS. Robot-assisted radical cystectomy with intracorporeal urinary diversion - the new ‘gold standard’? Evidence from a systematic review. Arab J Urol. 2018;16(3):307–13. 30140466 10.1016/j.aju.2018.01.006 PMC6104669

[4] Novara G, Catto JW, Wilson T, Annerstedt M, Chan K, Murphy DG, et al. Systematic review and cumulative analysis of perioperative outcomes and complications after robot-assisted radical cystectomy. Eur Urol. 2015;67(3):376–401. 25560798 10.1016/j.eururo.2014.12.007

[5] Lauridsen SV, Tønnesen H, Jensen BT, Neuner B, Thind P, Thomsen T. Complications and health-related quality of life after robot-assisted versus open radical cystectomy: a systematic review and meta-analysis of four RCTs. Syst Reviews. 2017;6(1):150. 10.1186/s13643-017-0547-y PMC5541663 28768530

[6] Visioni A, Shah R, Gabriel E, Attwood K, Kukar M, Nurkin S. Enhanced recovery after surgery for noncolorectal surgery? A systematic review and Meta-analysis of major abdominal surgery. Ann Surg. 2018;267(1):57–65. 28437313 10.1097/SLA.0000000000002267

[7] Cerantola Y, Valerio M, Persson B, Jichlinski P, Ljungqvist O, Hubner M, et al. Guidelines for perioperative care after radical cystectomy for bladder cancer: enhanced recovery after surgery (ERAS^®^) society recommendations. Clinical nutrition (Edinburgh. Scotland). 2013;32(6):879–87. 10.1016/j.clnu.2013.09.014 24189391

[8] Campbell KL, Winters-Stone KM, Wiskemann J, May AM, Schwartz AL, Courneya KS, et al. Exercise guidelines for Cancer survivors: Consensus Statement from International Multidisciplinary Roundtable. Med Sci Sports Exerc. 2019;51(11):2375–90. 31626055 10.1249/MSS.0000000000002116 PMC8576825

[9] McTiernan A, Friedenreich CM, Katzmarzyk PT, Powell KE, Macko R, Buchner D, et al. Physical activity in Cancer Prevention and Survival: a systematic review. Med Sci Sports Exerc. 2019;51(6):1252–61. 31095082 10.1249/MSS.0000000000001937 PMC6527123

[10] Schmitz KH, Courneya KS, Schneider CM, Von Gruenigen VE, Schwartz AL, Matthews C, et al. American College of Sports Medicine Roundtable on Exercise guidelines for Cancer survivors. Med Sci Sports Exerc. 2010;42(7):1409–26. 20559064 10.1249/MSS.0b013e3181e0c112

[11] Williams K, Steptoe A, Wardle J. Is a cancer diagnosis a trigger for health behaviour change? Findings from a prospective, population-based study. Br J Cancer. 2013;108(11):2407–12. 23695026 10.1038/bjc.2013.254 PMC3681023

[12] Koelker M, Alkhatib K, Briggs L, Labban M, Meyer CP, Dieli-Conwright CM, et al. Impact of exercise on physical health status in bladder cancer patients. Can Urol Association J = J de l’Association des urologues du Can. 2023;17(1):E8–14. 10.5489/cuaj.8008 PMC9872822 36121887

[13] Porserud A, Karlsson P, Rydwik E, Aly M, Henningsohn L, Nygren-Bonnier M, et al. The CanMoRe trial - evaluating the effects of an exercise intervention after robotic-assisted radical cystectomy for urinary bladder cancer: the study protocol of a randomised controlled trial. BMC Cancer. 2020;20(1):805. 32842975 10.1186/s12885-020-07140-5 PMC7448437

[14] Skivington K, Matthews L, Simpson SA, Craig P, Baird J, Blazeby JM, et al. A new framework for developing and evaluating complex interventions: update of Medical Research Council guidance. BMJ. 2021;374:n2061. 34593508 10.1136/bmj.n2061 PMC8482308

[15] Moullin JC, Sabater-Hernández D, Fernandez-Llimos F, Benrimoj SI. A systematic review of implementation frameworks of innovations in healthcare and resulting generic implementation framework. Health Res Policy Syst. 2015;13(1):16. 25885055 10.1186/s12961-015-0005-z PMC4364490

[16] Tong A, Sainsbury P, Craig J. Consolidated criteria for reporting qualitative research (COREQ): a 32-item checklist for interviews and focus groups. Int J Qual Health Care. 2007;19(6):349–57. 17872937 10.1093/intqhc/mzm042

[17] Braun V, Clarke V. Using thematic analysis in psychology. Qualitative Res Psychol. 2006;3(2):77–101.

[18] Rammant E, Fonteyne V, Decaestecker K, Bultijnck R, Deforche B, Pieters R, et al. Understanding physical activity behavior in patients with bladder cancer before and after radical cystectomy: a qualitative interview study. Clin Rehabil. 2019;33(4):750–61. 30514109 10.1177/0269215518815531

[19] Karvinen KH, Courneya KS, Venner P, North S. Exercise programming and counseling preferences in bladder cancer survivors: a population-based study. J Cancer Surviv. 2007;1(1):27–34. 18648942 10.1007/s11764-007-0010-5

[20] Porserud A, Sherif A, Tollbäck A. The effects of a physical exercise programme after radical cystectomy for urinary bladder cancer. A pilot randomized controlled trial. Clin Rehabil. 2014;28(5):451–9. 24249842 10.1177/0269215513506230

[21] Hirschey R, Bryant AL, Macek C, Battaglini C, Santacroce S, Courneya KS, et al. Predicting physical activity among cancer survivors: Meta-analytic path modeling of longitudinal studies. Health Psychology: Official J Div Health Psychol Am Psychol Association. 2020;39(4):269–80. 10.1037/hea0000845 PMC7203971 32011152

[22] McMullen CK, Kwan ML, Colwell JC, Munneke JR, Davis JV, Firemark A et al. Recovering from Cystectomy: Patient Perspectives. Bladder cancer (Amsterdam, Netherlands). 2019;5(1):51–61. 10.3233/BLC-180202 PMC6401661 30854413

[23] Yi Y, Yang Y, Shi X, Yang X. The unmet rehabilitation needs of colorectal cancer survivors after surgery: a qualitative meta-synthesis. Nurs open. 2024;11(1):e2051. 38268281 10.1002/nop2.2051 PMC10697127

[24] Guest G, Namey E, Taylor J, Eley N, McKenna K. Comparing focus groups and individual interviews: findings from a randomized study. Int J Soc Res Methodol. 2017;20(6):693–708.

[25] Malterud K, Siersma VD, Guassora AD. Sample size in qualitative interview studies:guided by Information Power. Qual Health Res. 2016;26(13):1753–60. 26613970 10.1177/1049732315617444

[26] Shenton AK. Strategies for ensuring trustworthiness in qualitative research projects. Educ Inform. 2004;22:63–75.

[27] Nowell LS, Norris JM, White DE, Moules NJ. Thematic analysis: striving to meet the trustworthiness Criteria. Int J Qualitative Methods. 2017;16(1):1–13.

