# Supplementary material for: Patients´ experiences of an exercise intervention in primary care following robot-assisted radical cystectomy due to bladder cancer: a qualitative study
Source: BMC Cancer. 2024 Oct 24;24:1306. doi: 10.1186/s12885-024-13059-y (PMC11515536; doi:10.1186/s12885-024-13059-y)
Supplement: Supplementary file 2 — Supplementary Material 2 [file 12885_2024_13059_MOESM2_ESM.docx]

# Interview guide

**Opening questions:**

Please tell us what it was like coming back home from the hospital.
Did you experience any difficulties in your everyday life because of the operation?

- In what way?
- What did it feel like physically?
- What did it feel like mentally?
- Were you able to do everything that you used to do before the operation?

**Starting the training:**

When you were discharged from the hospital, what thoughts did you have about beginning training with a physiotherapist?
How did the first contact with the physiotherapist happen?
Please describe your first meeting.
Did you feel that the physiotherapist had an understanding of you and your situation?

- What were your expectations of the training?
- Did it feel as if it was too soon/too late?
- How did the contact happen? Easy to contact/make an appointment? Attuned to possible worries/symptoms?

**The training:**

How many times did you do training with the physiotherapist?
Perhaps you could tell me what a typical training session might look like.
Please tell me how you and the physiotherapist collaborated during your sessions?
Do you feel that the training has made a difference? In what way?
Please tell me if you experienced any setback or complication in connection with the training.
Please tell me about something that felt particularly good during the time when you trained with a physiotherapist.
Something that felt less good.
Did you have any training goals of your own?

- In relation to expectations/aims?
- Good/bad collaboration?
- Feeling of progress?
- Difference between the sessions one time/another time, early in the process/later?

**Ending the training:**

How did the period of training with the physiotherapist end?
Have you continued with any training now, after you finished with the physiotherapist?
Please describe what your physical activity or day-to-day exercise looks like during an ordinary week now.
Has the training you did affected your everyday activities?

- What did it feel like to finish?
- Would you like to change anything?
- Feeling of physical functioning compared to before the operation? Before the training?

**Finally:**

Do you have any tips or pieces of advice that you would like to give to someone else who is about to start training after this type of operation?
Do you have any general feedback or tips that you would like to give to those physiotherapists who provide this training for patients who have had your type of operation?
Is there anything else that you would like to tell me about the training or your exercise that we have not asked you about?
